# The distinct role of orbitofrontal and medial prefrontal cortex in encoding impulsive choices in an animal model of attention deficit hyperactivity disorder

**DOI:** 10.3389/fnbeh.2022.1039288

**Published:** 2023-01-06

**Authors:** Aihua Cao, Dandan Hong, Chao Che, Xiaoxiao Yu, Zhifeng Cai, Xiaofan Yang, Di Zhang, Ping Yu

**Affiliations:** ^1^Department of Pediatrics, Qilu Hospital, Shandong University, Jinan, China; ^2^Beijing Key Laboratory of Learning and Cognition, College of Psychology, Capital Normal University, Beijing, China; ^3^Department of Neurosurgery, Qilu Hospital, Shandong University, Jinan, China

**Keywords:** ADHD, OFC, mPFC, neuronal discharge, delay discounting

## Abstract

Attention deficit hyperactivity disorder (ADHD) is a complex neurodevelopmental disorder affecting up to 5% of children worldwide. The lack of understanding of ADHD etiology prevented the development of effective treatment for the disease. Here, using *in vivo* electrophysiology recordings, we have recorded and analyzed the neuronal encoding of delay discounting behavior in prefrontal and orbitofrontal cortex of spontaneously hypertensive rat (SHR). We found that in the presence of rewards, neurons in the orbitofrontal cortex (OFC) were activated regardless to the value of the rewards and OFC neurons in SHR exhibited significantly higher rates of neuronal discharging towards the presence of rewards. While in the medial prefrontal cortex (mPFC), neurons of SHR responded similarly in the presence of large rewards compared with control rats whereas they displayed higher firing rates towards smaller rewards. In addition, the reward-predicting neurons in the OFC encodes for value of rewards in control animals and they were strongly activated upon receiving a small immediate reinforcer in the SHR whereas the reward-predicting neurons in the mPFC neurons generally did not respond to the value of the rewards. Our study characterized the neuronal discharging patterns of OFC and mPFC neurons in the SHR and the control animals and provided novel insights for further understanding the neuronal basis of ADHD pathology.

## Introduction

Attention deficit hyperactivity disorder (ADHD) is a neurodevelopmental disorder characterized by a persistent pattern of inattention, impulsivity, and locomotor hyperactivity affecting up to 5% of children worldwide (Polanczyk et al., 2015). Approximately 60% of clinically referred ADHD children will continue to have impairing symptoms as young adults (Faraone and Biederman, 2005; Faraone et al., 2006). Besides, there is an increased chance of developing psychiatric comorbidities, including depression, anxiety, and substance abuse (Klassen et al., 2004; Wehmeier et al., 2010).

In addition to executive function impairments, ADHD patients frequently exhibit abnormal motivation and reward processes, leading to impulsive behaviors. Impulsivity occurs due to either the attenuation of dopamine signaling to the appropriate thoughts and actions or deficits in goal-directed control of inappropriate behaviors (Pine et al., 2010). The prefrontal cortex, including the orbital frontal cortex (OFC) and dorsolateral prefrontal cortex (dlPFC), is essential for controlling goal-directed activity (Anonymous, 1994). Lesion and pharmacological inactivation of OFC or medial prefrontal cortex (mPFC; the rodent homolog of primate dlPFC based on the connectional and neuropsychological studies (Preuss, 1995; Bechara et al., 2000; Uylings et al., 2003; Vertes, 2006), can dramatically alter the impulsive choices in both humans and rodents (Preuss, 1995; Uylings et al., 2003; Berlin et al., 2004; Winstanley et al., 2004; Vertes, 2006; Churchwell et al., 2009; Macmaster and Rosenberg, 2010), suggesting its close involvement in regulating impulsive behaviors under multiple contexts (Bechara et al., 2000; Berlin et al., 2004; Churchwell et al., 2009; Gill et al., 2010; Mar et al., 2011). Regardless of the close involvement of the prefrontal cortex (PFC) in regulating delay discounting behavior, ADHD patients display disrupted structural and/or functional integrity of the PFC, including delayed maturation, hypoactivity, and altered frontostriatal connection (Fernandez et al., 2009; Cortese, 2012). Studies with an animal model of ADHD also revealed that disrupted glutamatergic transmission in PFC is responsible for ADHD-related behavioral deficits (Cheng et al., 2017). However, it is unclear whether and how the activity of PFC and its subregions are regulated in the pathological conditions of ADHD.

In both humans and animals, impulsivity choices are measured with a delay discounting task (DDT), allowing the subject to choose between a small immediate reward and a larger delayed reward. Previous studies have identified the activity patterns of distinct neuronal populations within the rat OFC and mPFC during discrete elements in the DDT (Roesch et al., 2006). In this study, we used the spontaneously hypertensive rats (SHR), which exhibit both behavioral deficits and genetic changes associated with human ADHD (Sagvolden et al., 2009; Meneses et al., 2011) as the animal model and performed electrophysiological recording in neurons of mPFC and OFC during a DDT. Rats were allowed to choose between a small immediate reward (one food pellet) vs. a large reward (three food pellets) available immediately or after a certain time of delay. Neuronal activity during cues predicting the rewards, anticipation of the large delayed rewards after lever pressing, and response to the actual rewards was recorded and analyzed. The results demonstrated that a subpopulation of OFC neurons in both SHR and Sprague-Dawley (SD) control rats responded to the reward-predicting cues and the value of the actual rewards despite whether there is a delay. Meanwhile, the activity of mPFC neurons only altered as the value of rewards changed. Interestingly, the activity of OFC neurons was significantly suppressed in SHR compared with the SD controls during anticipation of the large delayed rewards. These findings confirmed the role of OFC in encoding and processing the expectancy signal and cues for the delayed reward during delay discounting and impulsive choices, suggesting that the increased delay discounting in SHR could be induced by the hypofunction of OFC.

## Materials and Methods

### Subjects

Male Sprague Dawley (SD) rats and SHR (*n* = 8 each group), weighing 250–350 g, were purchased from the Shanghai SLAC Laboratory Animal Co. Rats were singly housed with food and water at the *libitum* under a standard 12 h:12 h light-dark cycle. During the behavioral tasks, rats were maintained at 85%–90% of pre-experimental body weights by food restriction. All animal experimental procedures were performed in accordance with the animal protocol approved by Local Committee of Animal Use and Protection at Capital Normal University.

### Surgery

Rats were deeply anesthetized by pentobarbital sodium (50 mg/kg, i.p.) before surgery. A 4 × 4 microwire electrode array made of 16 30 μm-diameter FeNiCr wires (California Fine Wire Co., Grover Beach, USA) was implanted in the OFC (AP +2.7–4.7, ML+ 2.7–3.7, DV 3.6–4.2 from bregma) and mPFC (AP+2.5–4.5, ML+0–1, DV 2.7–3.2 from bregma) region of the rat brain, respectively. Besides, anchoring screws were employed to anchor the electrode arrays and connect the electrode ground wire. Dental acrylic was adopted to further secure the electrode arrays and cover the ground wire. Rats were allowed to recover for at least 7 days before the experiments.

### Delay discounting behavioral task

Delay discounting, including the training phase and test phase, was performed in 30.5 × 24 × 21 cm Plexiglas chambers housed in a sound-attenuating cabinet (Med Associates). This was described in detail previously (Saddoris et al., 2011). The floor of the chamber is a grid consisting of stainless-steel rods (19.8 mm in diameter). Two retractable levers were equipped at the front wall of the chamber, with a distance of 10.5 cm above the grid floor. Food pellets were delivered to a food receptacle located in the middle of the two levers. A house light (100 mA) and a speaker were located at the back wall of the chamber providing the environmental light and the sound cue.

All behavioral tests were conducted at least 1 week after the electrode array implantation. Rats were first habituated with lever pressing. Pressing either one of the levers in the chamber led to a drop of a single food pellet. Rats reaching 50 times of lever-pressing on each lever within 30 min were selected for the DDT. The task contained three blocks. Each block was composed of 30 trials. The first 20 trials of each block were forced choices; the last 10 trials were free choices. In forced-choice immediate trials, a single visual cue above the designated lever was presented for 5 s before the extension of the lever. A single press of the left lever resulted in an immediate release of one food pellet. In forced-choice delay trials, the visual cue was presented before the extension of each lever. A single press of the right lever caused the release of three food pellets after a 0-s, 10-s, or 20-s delay, respectively on different trials. Pressing the lever with no visual cue associated was considered an omission. In the free-choice trials, both cue lights were turned on for 5 s, followed by the extension of both levers. Pressing one lever led to an immediate release of one food pellet and pressing the other level led to the release of three food pellets after the delay. For all delay discounting tasks, the choices were intermingled to avoid place preference. If animals failed to respond within 10 s, the trial was counted as an omission. After being trained on the task, the electrophysiological recording was performed on rats to determine the neuronal activity of OFC and mPFC during discrete task events.

### Electrophysiology recordings and neuron classification

Electrophysiological procedures have been described in detail previously (Hong et al., 2019). Extracellular unit activity was recorded using the multi-channel single units recording system (Cerebus, Blackrock Microsystem, UT, USA). Signals were acquired using a 16-channel digital headstage connected to the electrode array. Single unit data were separated through a high-pass digital filter (500–7,500 Hz), sampled at 30 kHz into the workstation computer, and then processed using Cerebus offline sorting algorithms, followed by manually re-sorting. Only the single-unit activity clearly separated from background noise was employed for analyses (Yin et al., 2009). Changes in neuronal firing relative to critical behavioral events were analyzed by constructing peri-event histograms surrounding each task event including baseline, cue presentation, lever press, and reward completion, with the bin width of 100 ms using Neuroexplorer5. Each cell was analyzed for changes in activity during cue presentation (0–5 s after cue presentation), lever pressing (0–5 s after lever pressing), and reward completion (0–5 s after response completion). Cells were classified as phasic if the firing frequency was significantly higher or lower than baseline (*p* < 0.05, two-way ANOVA with *post-hoc* Fisher’s LSD test) during one of the above events for at least one 100 ms time bin. All electrode positions were verified before data analysis to make sure the recordings were made from the right region (Supplementary Figure S1).

### Histology

Upon completion of the experiment, the placement of electrode tips was marked by passing DC (40-μA) through the recording electrodes for 20 s. Then, rats were deeply anesthetized with an overdose of pentobarbital (100 mg/kg, i.p.) and underwent transcardial perfusion with 100 ml of 0.02 M phosphate buffer and 100 ml of 4% paraformaldehyde solution. The brain was removed and sequentially transferred into 10 mM, 20 mM, and 30 mM sucrose solutions. After dehydrating, the brains were transferred into 4% paraformaldehyde for 3 days. Coronal sections at 50-μm thickness were cut on a freezing microtome (SM2010R, Leica, Nussloch, Germany). The sections were counterstained with Nissl. It was confirmed that the tips of the recording electrodes were located within the OFC and mPFC in all tested rats.

### Statistics

Statistical analysis of behavioral data was performed by SPSS19.0. For each session, the preference of choice was calculated as (trials for choosing big reward − trials for small reward)/total trials. Then, these values of all sessions for each rat were averaged as the final value of preference of choice for one rat. Concerning electrophysiology data, each neuron was analyzed in 100 ms bins with the bin and conditions as a repeated-measures factor (period) and an independent-measure factor, respectively. Statistical analysis of the firing frequency of recorded neurons was performed by SPSS19.0. All results were expressed as mean ± SEM values.

## Results

### Impulsive behaviors in SHR

**Figure 1:**
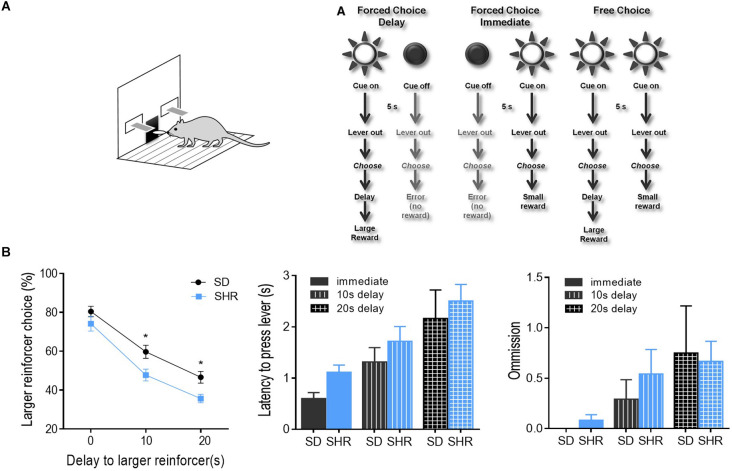
Percentage of large reinforcer choice in SD and SHR rats in delay discounting task. **(A)** Experimental design. Rats underwent 40 trials of forced choices (block 1 and 2) before being tested for 20 trials of free choices (block 3) for delay discounting. **(B)** Free choice behavior in SD and SHR rats during the delay discounting task with a delay time of 0, 10, and 20 s. The percentage of larger reinforcer choices decreased as the delay to reward increased in both SD and SHR rats while the SHR rats showed less preference to the larger reinforcer compared with SD rats (*p < 0.05). Data are presented as mean ± sem. SD, Sprague Dawley; SHR, spontaneously hypertensive rat.

Delay discounting behavior task was performed on the SHR rats and SD rats to test the degree of impulsivity in SHR rats. The experimental procedure was illustrated in Figure 1A. As shown in Figure 1B, both groups of rats displayed classic delay discounting behavior. Both rats displayed classic delay discounting behavior. During the free choice trials, preference for the larger reward (three pellets) decreased as the delay in obtaining this outcome increased in both SD and SHR rats. However, SHR rats presented a significantly lower percentage of large reinforcer choices compared with SD rats when the delay time increased. The two-way ANOVA (group × delay) revealed a main effects of group (*F*_(1,14)_ = 6.036, *p* = 0.0277) and delay to the large reward (*F*_(2,28)_ = 296.6, *p* < 0.0001), but no interaction between group and delay time (*F*_(2,28)_ = 2.048, *p* = 0.1479). Additionally, no difference in latency to lever press (two-way ANOVA, group: *F*_(1,14)_ = 2.611, *p* = 0.114; delay: *F*_(2,28)_ = 10.792, *p* < 0.001; interaction: *F*_(2,28)_ = 0.0382, *p* = 0.963) and omissions (two-way ANOVA, group: *F*_(1,14)_ = 0.175, *p* = 0.678; delay: *F*_(2,28)_ = 3.758, *p* = 0.032; interaction: *F*_(2,28)_ = 0.234, *p* = 0.793) between the two groups of rats was observed (Figure 1B). These results demonstrated that SHR rats choose more impulsively than SD rats on the DDT.

### The activity of reward-responding neurons in OFC of SHR and SD rats

**Figure 2:**
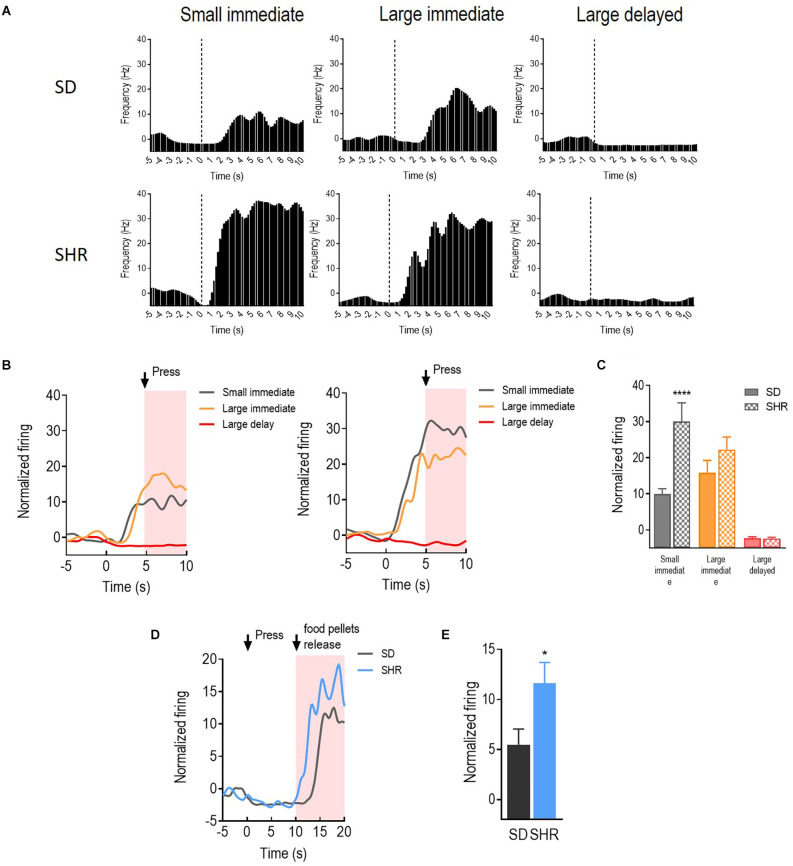
The orbitofrontal cortex (OFC) neurons respond to reward in SD and SHR rats. **(A)** Peri-event histograms of representative OFC neurons in SD rats (upper panel) and SHR rats (lower panel) in response to the sized reinforcer. Data are aligned to reward onset (lever pressing at time 0, dashed line). **(B)** Normalized firing response of all OFC neurons response to sized reinforcer in SD rats (left panel) and SHR (right panel) rats. **(C)** Quantification of the normalized firing rate of OFC neurons in both SD and SHR rats during the 5 s period after the lever press (highlighted box). **(D)** Normalized firing rate of all OFC neurons’ response to large delayed reinforcer in SD and SHR rats. **(E)** Quantification of the normalized firing rate of OFC neurons in both SD and SHR rats during the 5 s period after the release of the large delayed reinforcer. *p < 0.05, ****p < 0.0001 compared with SD rats.

With the behavioral difference observed in SHR rats, we further investigated the underlying electrophysiological mechanisms involved in regulating delay discounting behavior. The total number of 114 and 126 responding neurons were recorded in the SD and SHR, respectively. The numbers of event-responding neurons in mPFC and OFC were listed in Table 1. The reward-responding neurons in OFC of both SD and SHR rats were first examined in all tested blocks. In all recorded neurons, a total number of 63 OFC neurons recorded from 16 animals (*n* = 8 SD; *n* = 8 SHR) exhibited clear phasic changes in firing rate after the reward-completion stage in the DDT. A representative reward-responding neuron in OFC of SD rats and SHR rats was illustrated in Figure 2A (upper: SD, lower: SHR). The average firing rate of all reward-responding neurons in OFC of SD rats and SHR rats was exhibited in Figure 2B (left: SD, right: SHR). The reward-responding neurons in the OFC of both SHR and SD control rats revealed increased firing rates in the presents of the rewards when the firing rates within 5 s after lever pressing were analyzed (Figure 2B left and middle). The two-way ANOVA analysis suggested significant difference in normalized firing rates between SD and SHR (groups: (*F*_(1,19)_ = 8.631, *p* = 0.008); value of the reinforcer (*F*_(2,38)_ = 54.45, *p* < 0.001); group × value of the reinforcer (*F*_(2,38)_ = 9.105, *p* < 0.001). *Post-hoc* analysis (Bonferroni correction) demonstrated significantly higher firing rates of the OFC neurons in the SD rats responding to the small immediate reinforcer and the large immediate reinforcer in comparison with which to the large delayed reinforcer during the period when there was no reinforce (small immediate vs. large delayed: *p* = 0.02; large immediate vs. large delayed: *p* < 0.001). Meanwhile, there was no difference in the firing rates between the small immediate and large immediate reinforcers (*p* = 0.255). The OFC neurons in the SHR rats responded to the different reinforcers in a similar way as that of the SD rats (small immediate vs. large delayed: *p* < 0.001; large immediate vs. large delayed: *p* < 0.001; small immediate vs. large immediate: *p* = 0.095). However, the OFC neurons in the SHR rats exhibited significantly higher firing rates in responding to small immediate rewards compared to that of SD rats (SD vs. SHR within small immediate reinforcer: *p* < 0.001; Figure 2C). The increased firing rates of OFC neurons in the SHR rats were not induced by a general overactivation of OFC in these animals because they were not different in the two groups during the time before the food was released. We further analyzed the normalized firing rates of OFC neurons in responding to the large delayed reinforcer after the food pellets were released to the animals (within 10 s) and the OFC neurons in the SHR rats also showed an increased firing rate (*p* = 0.0285; Figures 2D,E). These results demonstrated that OFC neurons are activated in responding to rewards regardless of the value of the reinforcer, and the OFC neurons in the SHR rats respond more vigorously to rewards.

**Table 1:** Neuronal firing patterns in Sprague Dawley (SD) rat and spontaneously hypertensive rat (SHR).

	**SD**		**SHR**	
	**mPFC**	**OFC**	**mPFC**	**OFC**
Reward-responding neurons	36 (15%)	33 (13.75%)	30 (12.5%)	30 (12.5%)
Reward-predicting neurons	18 (7.5%)	27 (11.25%)	21 (11.25%)	45 (18.75%)
Total	114	126

### The activity of reward-responding neurons in mPFC of SHR and SD rats

**Figure 3:**
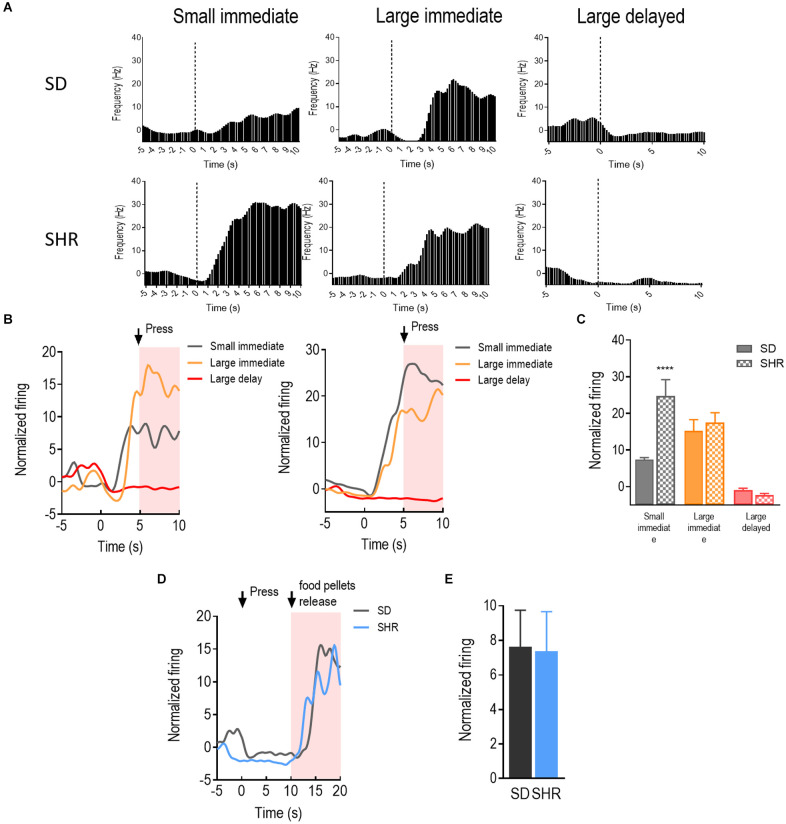
The medial prefrontal cortex (mPFC) neurons respond to reward in SD and SHR rats. **(A)** Peri-event histograms of representative mPFC neurons in SD rats (upper panel) and SHR rats (lower panel) in response to the sized reinforcer. Data are aligned to reward onset (lever pressing at time 0, dashed line). **(B)** Normalized firing rate of all mPFC neurons in response to sized reinforcer in SD rats (left panel) and SHR (right panel) rats. **(C)** Quantification of the normalized firing rate of mPFC neurons in both SD and SHR rats during the 5 s period in response to the lever press (highlighted box). **(D)** Normalized firing rate of all mPFC neurons’ response to large delayed reinforcer in SD and SHR rats. **(E)** Quantification of the normalized firing rate of mPFC neurons in both SD and SHR rats during the 5 s period after the release of the large delayed reinforcer. ****p < 0.05 compared with SD rats.

With a similar strategy, the reward-responding neurons in mPFC of both SD and SHR rats were further examined. A total number of 66 mPFC neurons were recorded and analyzed from 16 animals (*n* = 8 SD; *n* = 8 SHR) during the DDT. The representative reward-responding neurons and the normalized firing rates were illustrated in Figures 3A,B. We found that the reward-responding neurons in the mPFC also presented significantly higher firing rates in the present of rewards in both SD and SHR rats (group: *F*_(1,20)_ = 6.279, *p* = 0.021); value of reinforcer: (*F*_(2,40)_ = 49.835, *p* < 0.001); group × value of reinforcer interaction: (*F*_(2,40)_ = 11.469, *p* < 0.001). Thus, mPFC neurons are generally responsive to reward in both SD and SHR rats. *Post-hoc* analysis revealed similar responses observed with the rewarding neurons in the OFC. The rewarding neurons in the mPFC of SHR rats showed a significantly higher firing rate upon receiving the small immediate reinforcer compared with the SD rats (*p* < 0.001; Figure 3C). Simultaneously, there was no difference observed when rats received large immediate rewards between the two groups. The normalized firing rates of recorded neurons in the group of rats that received large delayed rewards (10 s after lever pressing) were further analyzed within 10 s after reward release in SD and SHR rats (Figures 3D,E). No difference in firing rate between the two groups was observed (*t*_(20)_ = 0.077, *p* = 0.9394). These results suggested that the mPFC neurons in the SHR rats responded normally to large reinforcers compared with SD rats while they are more active in response to the small immediate reinforcer.

### The activity of reward-predicting neurons in OFC of SHR and SD rats

**Figure 4:**
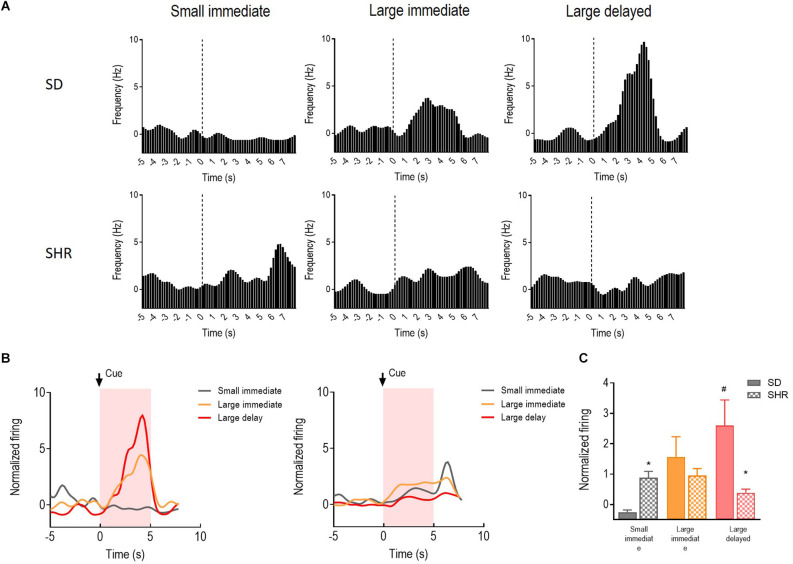
The OFC neurons respond to reward-predicting cues in SD and SHR rats. **(A)** Peri-event histograms of representative OFC neurons in SD rats (upper panel) and SHR rats (lower panel) in response to the reward-predicting cue. Data are aligned to cue onset (cue sound starts at time 0, dashed line, last for 3 s). **(B)** Normalized firing response of all OFC neurons’ response to sized reinforcer in SD rats (left panel) and SHR (right panel) rats. **(C)** Quantification of the normalized firing rate of OFC neurons in both SD and SHR rats during the 3 s period of cue presentation (highlighted box). *p < 0.05 compared with SD rats; ^#^*p* < 0.05 compared with small immediate reinforcer within SD rats.

The reward-responding neurons in the OFC were recorded and analyzed to illustrate whether OFC neurons in SHR rats are deficient in encoding the reward expectation. A total number of 72 OFC neurons recorded from 16 animals (*n* = 8 SD; *n* = 8 SHR) exhibited clear phasic changes in firing rate to the reward-predicting cues during the DDT. Representative cue-responding neurons in response to the reward-predicting cue in OFC of SD rats and SHR rats were illustrated in Figure 4A (upper: SD, lower: SHR). The average firing rate of the cue-responding neurons in OFC of SD rats and SHR rats was depicted in Figure 4B (left: SD, right: SHR). Firing rates of the OFC neurons were analyzed for 5 s, during which the reward-predicting cues were presented. The two-way ANOVA analysis revealed a main significant effect of the interaction between the group and the value of the reinforcer (group: *F*_(1,22)_ = 1.738, *P* = 0.201, value of the reinforcer: *F*_(2,44)_ = 10.267, *p* < 0.001; group × value of the reinforcer: *F*_(2,44)_ = 18.347, *P* < 0.001). *Post-hoc* analysis demonstrated a significantly increased firing rate in the OFC cue-responding neurons in response to the delayed large reinforcer when compared to the small reinforcer in the SD rats (small vs. larger delayed: *p* < 0.001). Such effects were not observed in SHR rats (small vs. large delayed: *p* = 0.296). By comparing the firing rates between groups, an increased firing of the OFC cue-responding neurons in the SHR rats was observed during the expectation of a small immediate reinforcer (SD vs. SHR within small immediate reinforcer: *p* = 0.038) and a decreased firing during the expectation of a large delayed reinforcer in comparison with SD rats (SD vs. SHR within large delayed reinforcer: *p* < 0.001; Figure 4C). These results revealed that the activity of the cue-responding neurons in the OFC increased with the value of the reinforcer expected in the SD rats while the cue-responding neurons in the SHR rats were strongly activated upon receiving a small immediate reinforcer.

### The activity of reward-predicting neurons in mPFC of SHR and SD rats

**Figure 5:**
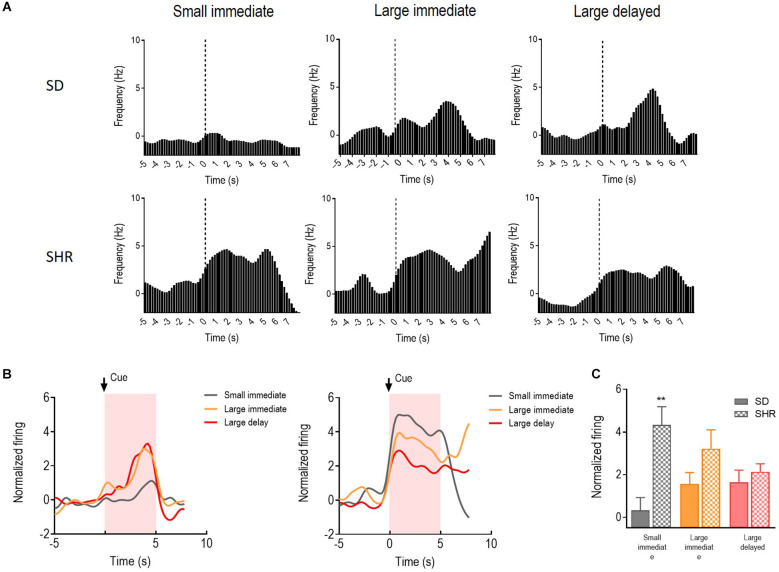
The mPFC neurons respond to reward-predicting cues in SD and SHR rats. **(A)** Peri-event histograms of representative mPFC neurons in SD rats (upper panel) and SHR rats (lower panel) in response to the reward-predicting cue. Data are aligned to cue onset (cue sound starts at time 0, dashed line, last for 3 s). **(B)** Normalized firing response of all mPFC neurons’ response to sized reinforcer in SD rats (left panel) and SHR (right panel) rats. **(C)** Quantification of the normalized firing rate of mPFC neurons in both SD and SHR rats during the 3 s period of cue presentation (highlighted box). **p < 0.01 compared with SD rats.

Activities of the 39 cue-responding neurons in the mPFC were recorded and examined during the 5 s when the reward predicting cue was presented in the DDT. The representative neuronal firing and the average firing rate were illustrated in Figures 5A,B. There was a main significant effects of the group (*F*_(1,11)_ = 6.511, *p* = 0.027) and the group × value of the reinforcer interaction between SD and SHR rats (*F*_(2,22)_ = 7.635, *p* = 0.003). Meanwhile, differences in firing rates of the mPFC cue-responding neurons were observed in the process of comparing animals during the expectation of the small immediate, large immediate, or large delayed reinforcers in SD or SHR rats. However, SHR rats exhibited a generally and significantly elevated neuronal activity when animals were expecting immediate rewards, regardless of the value of reinforcer (SD vs. SHR within small immediate: *p* < 0.001; SD vs. SHR within large immediate: *p* = 0.004; Figure 5C). These results confirmed that the activity of cue-responding neurons in the mPFC of the SD rats did not react to either the value or the delay of the rewards, and these neurons were generally more sensitive in SHR rats for immediate rewards.

## Discussion

The elevated impulsivity is often observed in patients with ADHD yet the neuronal basis of which has not been clearly illustrated. In the current study, we have recorded the discharge of neurons encoding reward and expected reward in OFC and mPFC cells of SHR and SD rats. We found that corresponding with the increased impulsivity in SHR rats, the neurons in OFC and mPFC of the SHR rats fired at significantly higher rates in either the presence of the small immediate rewards or the cue associated with it when compared with that of SD rats. The OFC neurons also showed reduced firing rates to the cue associated with large delayed rewards. These results provided the electrophysiological basis underlying the higher delay discounting behaviors in the model of ADHD rats.

The impulsivity behaviors of SHR rats were tested using the DDT, which measures the decline in response to the value of a reward with delay to its receipt. It is a frequently used experimental paradigm to test the impulsive behavior in both rodents and humans (Bailey et al., 2021). Using DDT, we found that both SHR and SD rats showed typical delay discounting behaviors. During the free choice trials, both SD and SHR rats showed a reduction in preference for the big rewards as the delay to obtain such rewards increased, however, the rate of delay discounting was significantly faster in the SHR rats, indicating the increase in impulsivity in the SHR rats. The SHR has been considered as a suitable rodent model of ADHD because they exhibit several behavioral characteristics often seen in ADHD patients, including impulsivity, impaired sustained attention, and hyperactivity (Sagvolden, 2000; Fox et al., 2008; Sontag et al., 2010). Consistent with our findings, Aparicio et al. (2019) have reported that SHR chooses even more impulsively than Wistar-Kyoto rats, which is another commonly used strain to study impulsivity (Adriani et al., 2003; Hand et al., 2009). In addition, the impulsive behaviors we observed in the SHR also mimicked the phenomenon of delay discounting in ADHD children who often prefer the small immediate rewards to the large delayed rewards (Blume et al., 2019; Mies et al., 2019).

Since the SHR exhibited impulsive behaviors, we next use them to further investigate the possible neuronal basis underlying the impulsivity in ADHD. Using *in vivo* electrophysiological recording, we first examined the neuronal discharge of OFC in SHR rats. The neuronal activities during the reward period and reward anticipation period in SHR and control SD rats were recorded and analyzed. Through analyzing the neuronal activities during the reward period, we found that both the SD and SHR rats showed significant increase in firing rate in their OFC neurons in the present of the reward. However, the firing frequencies in the OFC in response to reward did not seem to differ from the small to large reward. In addition, the neuronal activities were significantly higher in the OFC of SHR in response to the small reward. Since the OFC is a key brain region that encodes the value of rewards (Schoenbaum et al., 2003; Ostlund and Balleine, 2007; Howard and Kahnt, 2017; Malvaez et al., 2019; Rolls, 2019; Setogawa et al., 2019), our results indicate that the overaction of OFC in the presence of the small reward in the SHR might contribute to its impulsive behavior seen in the DDT. In support of our findings, ADHD children also exhibited abnormal activation of OFC. Studies using functional magnetic resonance imaging reported that compared with the normal control group, the OFC of ADHD children was abnormally activated in reward-related activities, the activation degree was highly correlated with high impulsivity/hyperactivity of ADHD, and high cognitive skills had a relationship with normal OFC response (von Rhein et al., 2015; Tegelbeckers et al., 2018). These results suggested that the overactivation of the OFC neurons in responding to the small immediate reward could over-emphasize the value of small immediate rewards which possibly led to the preference to small immediate reward in SHR rats during DDT. Other than value encoding, OFC neurons also signal the outcomes associated with particular stimuli (Stalnaker et al., 2014; Howard et al., 2015; Chen et al., 2018). We then analyzed neuronal activities of OFC in SHR and SD rats in response to the specific cue associated with different rewards during the anticipation period. We found that the reward-predicting neurons of SD rats’ OFC showed significant discharge when the rats anticipated the large rewards. However, this significant discharge for large rewards was absent in SHR rats. Comparatively, the reward-predicting neurons in the OFC of SHR rats exhibited significantly higher discharge responses to small rewards compared to that of SD rats. This implied that the reward-predicting neurons in the OFC of SHR rats were more sensitive to the anticipation of small rewards instead of large rewards. Categorical regression test using neuronal discharge as the dependent variable and behavioral choices as the independent variable further revealed statistically significant correlation between the neuronal discharging and the behavioral responses in both the reward-responding neurons and reward-predicting neurons in the OFC (Supplementary Table 1). Altogether, the over-discharge of reward neurons as well as reward-predicting neurons in the OFC of SHR to the small immediate rewards could possibly explain the increased rate of delay discounting we observed in the SHR.

In addition to the orbitofrontal lobe, mPFC also plays an essential role in value assessment, cognitive control, and imagination/expectation. We next determined the neuronal activities of mPFC neurons in SHR and SD rats. In SD rats, we observed the significant discharges of mPFC reward neurons in response to reward-receipt and the discharging frequency was proportionally increased with the value of the reward. These results were in line with several studies in both rodents and primates confirming that mPFC represents values of the reward (Pratt and Mizumori, 2001; Hayden and Platt, 2010; Cowen et al., 2012). The categorical regression test we performed also showed significant correlation between the neuronal discharge of mPFC reward-responding neurons and behavior choices, further confirming that the impact of these neurons on the behavior choices (Supplementary Table 1). However in the SHR, we observed a decreased firing frequency with the increase of value of the reward, implying that the dysfunction in the reward value encoding of mPFC reward neurons could also contribute to the behavioral choices toward the small immediate reward in the SHR. As to the reward-predicting neurons in the mPFC, we observed similar results. The proportional increase in the neuronal discharge in the reward-predicting neurons in response to the increase in reward value was missing in the SHR, indicating the lack of value-predicting encoding in the mPFC of SHR. In addition, the fact that the neuronal discharges in the reward-predicting neurons in the mPFC of SHR were significantly higher than that of the SD rats indicated that these neurons were more sensitive to small immediate rewards instead. Although we did not observe significant correlation between the neuronal discharge of reward-predicting in the mPFC and the behavior choices possibly due to small *n* number of neurons recorded (Supplementary Table 1), literatures suggested the important role of mPFC in regulating delay discounting behaviors (von Rhein et al., 2015). In support to our findings, imaging studies reported that human mPFC is activated when the subjects perform delay discounting tasks (Peters and Buchel, 2011; Fobbs and Mizumori, 2017). Moreover, the delay discounting level of human beings can be affected by simulating mPFC with transcranial magnetic stimulation (TMS; Cho et al., 2015). The inactivation or injury of mPFC can enhance delay discounting (Churchwell et al., 2009). A recent study using DDT revealed that the mPFC of rats can represent delayed changes within a short time, and the difference of rats in behavioral impulsivity can be reflected by the difference in mPFC activity to a certain extent (Peters and Buchel, 2011; Fobbs and Mizumori, 2017). Furthermore, the presence of functional impairment of mPFC in ADHD patients has been regarded as one of the possible reasons for the dysfunction and decision error in ADHD (Silvetti et al., 2014; Chantiluke et al., 2015). This region has always been considered related to neurological abnormalities during cognitive tasks in ADHD. The categorical regression test showed significant correlation between the neuronal discharge of mPFC reward-responding neurons and behavior choices and taken together, our findings further confirmed that the abnormal neuronal activities in mPFC might result in abnormal value assessment and anticipation for rewards in the SHR and subsequently attribute to the impulsive choices.

In summary, this study for the first time illustrated the neuronal discharging patterns of OFC and mPFC neurons in SHR towards rewards as well as during anticipation of rewards and revealed that the mal-activation in the OFC and mPFC neurons towards small immediate rewards could be the neuronal basis underlying the impulsivity in ADHD.

## Data Availability Statement

The raw data supporting the conclusions of this article will be made available by the authors, without undue reservation.

## Ethics Statement

The animal study was reviewed and approved by Local Committee of Animal Use and Protection at Capital Normal University.

## Author Contributions

AC, DZ, and PY: conceptualization. DH, XY, and CC: methodology. DH, XY, and ZC: data curation. AC and DZ: validation, writing—review and editing. AC and DZ: writing—original draft preparation. DZ: project administration. AC and PY: funding acquisition. All authors contributed to the article and approved the submitted version.
