# Supplementary material for: The distinct role of orbitofrontal and medial prefrontal cortex in encoding impulsive choices in an animal model of attention deficit hyperactivity disorder
Source: Front Behav Neurosci. 2023 Jan 6;16:1039288. doi: 10.3389/fnbeh.2022.1039288 (PMC9859629; doi:10.3389/fnbeh.2022.1039288)
Supplement: Supplementary file 1 [file Table_1.pdf]

Supplementary Table 1

Supplementary Table 1 Categorical regression and significance coefficients				
	β	df	F	P
Reward-responding neurons (OFC)	0.499	2	31.153	<0.0001
Reward-predicting neurons (OFC)	0.348	2	14.412	<0.0001
Reward-responding neurons (mPFC)	0.686	2	71.825	<0.0001
Reward-predicting neurons (mPFC)	0.077	2	0.514	0.602
