# Supplementary figures and images for: The distinct role of orbitofrontal and medial prefrontal cortex in encoding impulsive choices in an animal model of attention deficit hyperactivity disorder
Source: Front Behav Neurosci. 2023 Jan 6;16:1039288. doi: 10.3389/fnbeh.2022.1039288 (PMC9859629; doi:10.3389/fnbeh.2022.1039288)

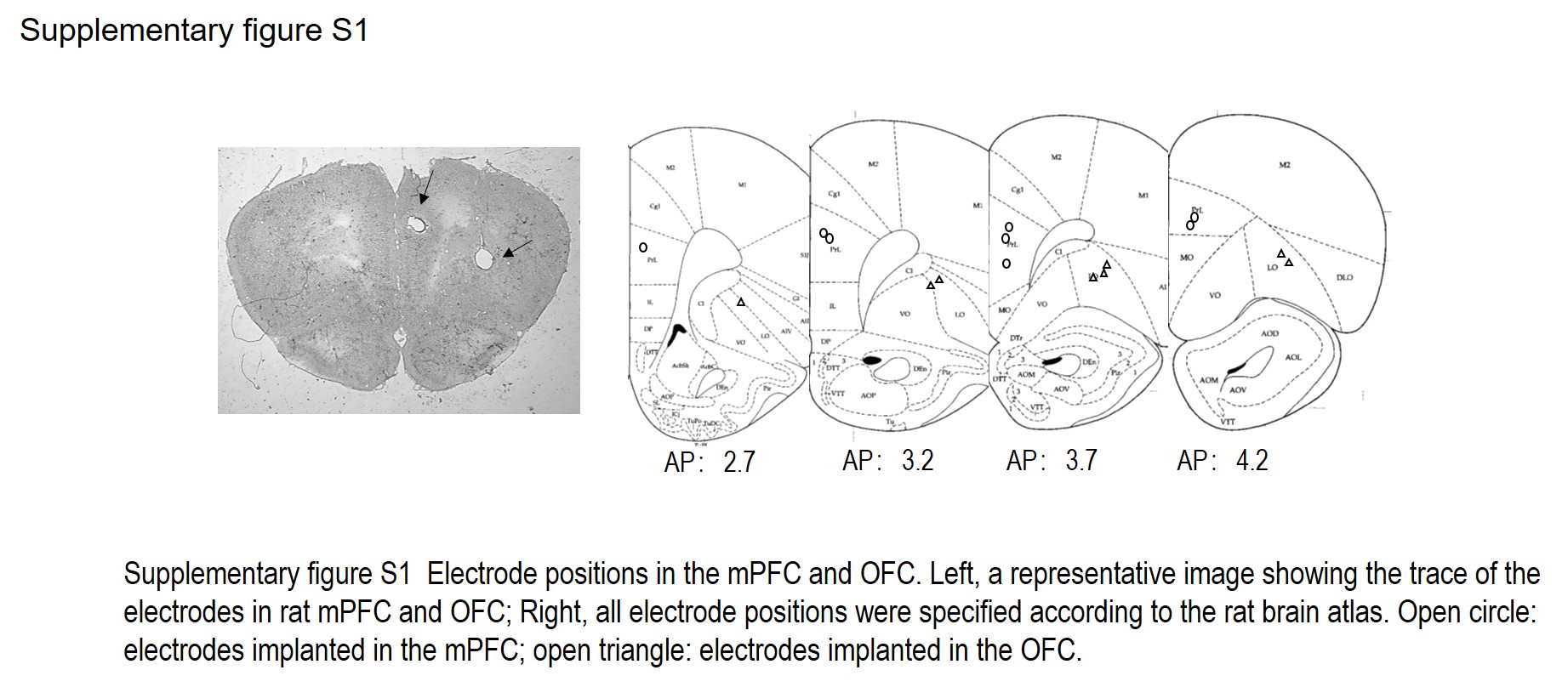

Supplement: Supplementary file 2 [file Image_1.JPEG]
